# Degradation of a mixture of 13 polycyclic aromatic hydrocarbons by commercial effective microorganisms

**DOI:** 10.1515/biol-2022-0831

**Published:** 2024-02-05

**Authors:** Paulina Książek-Trela, Damian Figura, Dominika Węzka, Ewa Szpyrka

**Affiliations:** Department of Biotechnology, Institute of Biotechnology, University of Rzeszow 1 Pigonia St., 35-310 Rzeszow, Poland

**Keywords:** polycyclic aromatic hydrocarbons, effective microorganisms, bioremediation, biodegradation, mixed chemicals

## Abstract

The study focused on the contribution of effective microorganisms (EM) and their consortia, used in commercial biological preparations and formulations for soil revitalization, to the degradation of a mixture of 13 polycyclic aromatic hydrocarbons (PAHs) commonly found in the soil environment. PAHs, diverse forms of which are present in the environment, never occur individually but always as a part of a chemical mixture. Therefore, the research presented in this article, focusing on the EM impact on the mixture of PAHs, reflects the conditions most similar to natural ones. On Day 35 of the experiment, PAH levels decreased by 75.5–95.5%. The highest PAHs degradation efficiency was achieved for fluorene, with a preparation containing eight bacteria strains from the *Bacillus* genus: *B. coagulans, B. amyloliquefaciens, B. laterosporus, B. licheniformis, B. mucilaginosus, B. megaterium, B. polymyxa,* and *B. pumilus*. All tested preparations containing bacterial consortia and a preparation with the yeast *S. cerevisiae* intensified the PAHs degradation more effectively than formulations including only the yeast *Yarrowia lipolytica* or a mixture of *Debaryomyces hansenii* and *Bacillus*. The designed and proposed research will contribute to the development of biotechnological methods – bioremediation by microorganisms that are safe for the human and environment health.

## 1 Introduction

**Figure 1:**
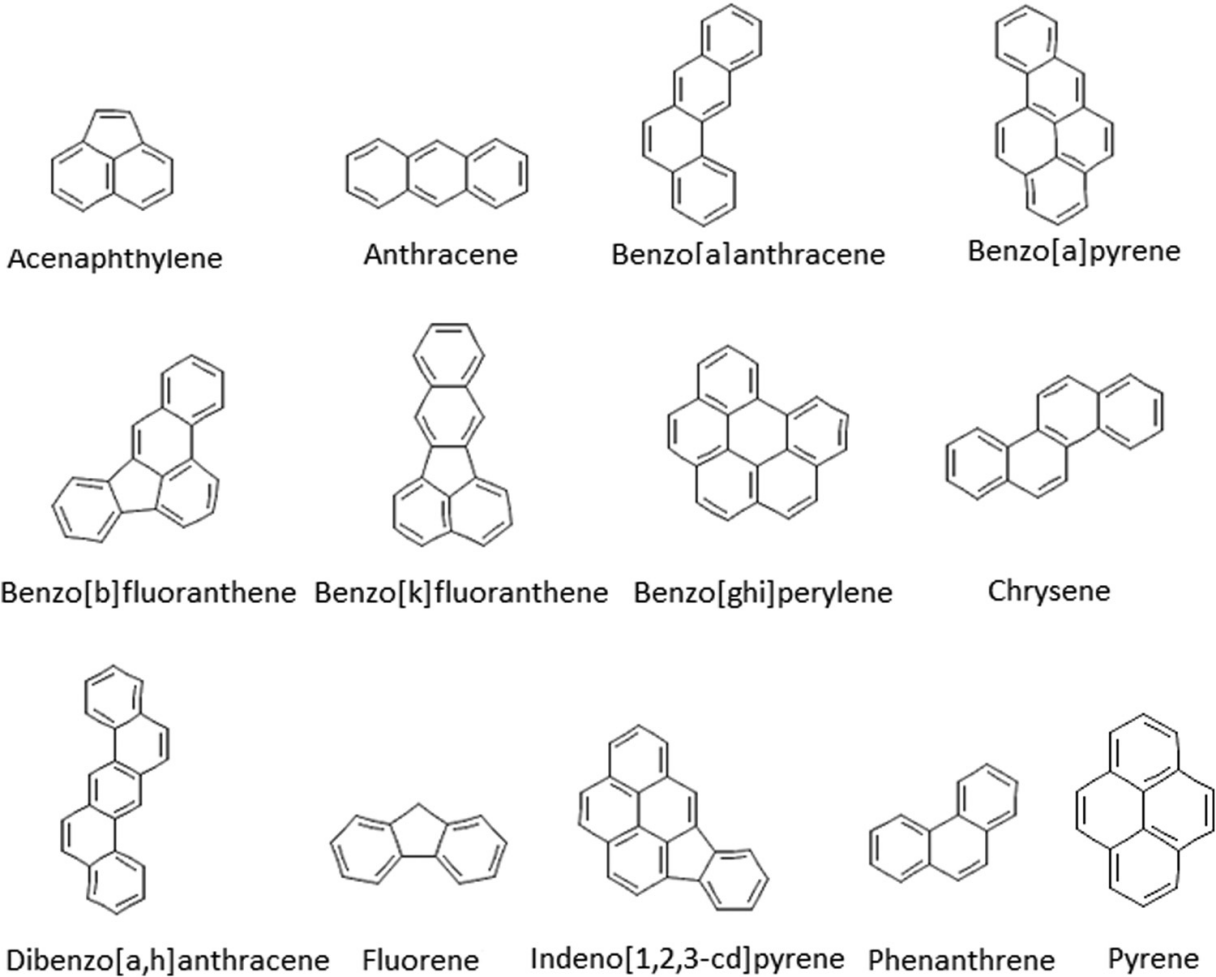
Names and chemical structures of studied PAHs [4].

Polycyclic aromatic hydrocarbons (PAHs) are a group of organic compounds that contain at least two joined aromatic rings of a flat surface structure (Figure 1) [1]. They form a group consisting of several hundred chemically related compounds, persistent in the environment, of variable toxicity, and having different structures [2]. PAHs are characterized by their carcinogenic, toxic, and mutagenic effects, they are also very strong immunosuppressants. It is thought that their toxicity mechanism is based on the fact that they disturb functions of cell membranes and enzymatic systems [3].

PAH are relatively inert hydrophobic compounds that in mammals can be transformed in metabolic processes into highly reactive dihydrodiol epoxides. These diol epoxides react with single strand and double strand DNA, and they may intercalate between base pairs [5]. PAHs can originate from natural and artificial sources, such as oil and gas. PAHs are produced as a result of biological processes and can be formed as a product of burning organic matter, such as wood and food [6]. PAHs are considered to be omnipresent in our environment and are found in soil, water, atmosphere, and air, as well as during food processing and cooking [1,7]. The sources of PAHs found in the soil include, for example, vehicle exhaust gases. Some PAHs come from more distant sources and were transported in the air to their final destination. PAHs at increased concentrations or at levels exceeding specified limits (amounting to 2.979 mg/kg) are observed in soil samples collected in the vicinity of transportation routes and in locations affected by tourist traffic and transport [8]. The majority of PAHs in the soil are bound with its particles, and this influences their mobility. Compounds of a low molecular mass are characterized by their relative mobility in the soil and bioavailability to soil microorganisms. However, it is the compounds of a high molecular mass that are more hazardous, because they are stable and hydrophobic, as well as have a low solubility in water [3,9]. Also, the soil properties influence PAHs susceptibility to sorption on the soil particles. Soil conductivity is an important factor influencing PAHs mobility [10]. These compounds are classified as persistent environmental pollutants, and they often occur as a mixture [11].

PAHs are removed from the environment by several methods, including biodegradation, photochemical degradation, photooxidation, washing out, bioaccumulation, or adsorption. Individual processes influence PAHs in different ways, as each compound has a unique structure and different chemical, biological and physical properties [2,12]. Biodegradation of persistent pollutants and soil bioremediation belong to those biotechnological interventions that are most advantageous, in terms of economic and environmental aspects [13–17]. Biodegradation is a preferred and main way for removing PAHs from polluted environments, because it is cost effective and enables complete removal of those compounds [18]. During last decades, a great progress has occurred in studies on PAHs bioremediation [19]. This process involves anaerobic or aerobic biochemical decomposition of organic compounds into simple inorganic compounds by saprobionts, such as bacteria and fungi, but also by yeasts, algae, and protozoa. The bacteria are regarded as key microorganisms for the PAHs degradation [20,21]. Usually, microorganisms are not able to directly decompose PAHs, so the microflora present in a given environment needs to adapt to the PAHs degradation. This is particularly important, taking into account low PAHs solubility in water that results in their low bioavailability to microorganisms [3]. Microorganisms also need time to develop the ability to produce necessary enzymes, depending on a type of microorganisms and properties of PAHs [22]. An individual bacterium or fungus is not able to degrade all contaminations. Thus, biodegradation is a multi-stage process occurring with a support of a large number of microorganisms that act synergistically [23]. PAHs of a low molecular weight (containing two or three fused benzene rings) can be easily degraded by microorganisms. PAHs of a high molecular weight, containing at least four rings, are resistant to biodegradation; therefore, they accumulate in the ecosystem [19,24,25]. Microorganisms degrading PAHs can metabolize PAHs as a sole source of energy and carbon in the soil [26].

Many studies on PAHs biodegradation focus on bacteria from genera *Nocardia, Pseudomonas, Gordonia, Micrococcus, Rhodococcus, Arthrobacter, Mycobacterium, Flavobacterium, Corynebacterium, Klebsiella, Alcaligenes,* and *Bacillus*, as well as fungi from genera *Penicillium, Aspergillus, Trichoderma, Candida,* and *Fusarium* [27–31]. While there are many reports on the PAHs degradation by microorganisms, including bacteria and fungi, the publications reporting the successful degradation of PAHs using yeasts are relatively scarce. The effect of yeasts on the degradation of crude oil or PAHs from petroleum-contaminated sites was analyzed in a few studies on *Saccharomyces cerevisiae, Yarrowia lipolytica*, *Hanseniaspora valbyensis, H. opuntiae,* and *Debaryomyces hansenii* [32–35]. Abioye and Ferreira [32,33] found that yeasts are effective in biodegradation of crude oil, while Mandal [34,35] analyzed decomposition of two PAHs –  – benzo[*a*]pyrene and benzo[*ghi*]perylene by a yeast consortium, and its effectiveness was at a level of 76 and 64%, respectively.

PAH bioremediation involves enzymes from bacteria, fungi, yeasts, and other living organisms. Biodegradation using enzymes is efficient and selective, due to higher reaction rates and the capability to catalyze reactions at a wide range of temperatures and pH values [3]. Enzymes responsible for the PAHs degradation include oxygenase, dehydrogenase, lignin peroxidase, manganese peroxidase, laccases, and phenoloxidases [36,37]. Dehydrogenase is an enzyme found in all viable microbial cells. Its activity is a measure of the metabolic state of soil microorganisms [38]. The dehydrogenase activity (DHA) is one of the most adequate, important, and sensitive bioindicators, related to the soil fertility [39]. This activity depends on the same factors that influence the abundance and activity of microorganisms. Besides, it is well known that pesticides, PAHs, and other persistent soil pollutants inhibit DHA [39–41]. Several environmental factors such as the soil moisture content, the oxidation–reduction potential (ORP), the pH, the temperature, the organic matter content, contamination with PAHs or pesticides, and soil fertilization, can significantly affect DHA in the soil [39]. The ORP is an important environmental factor, which reflects the tendency of an environment to receive or supply electrons in a solution [42]. ORP plays a crucial role in regulating the microbial activity, and affects the soil enzymatic activity, especially DHA [43]. Generally, the activity of enzymes tends to increase with the soil pH [44,45]. It was shown that the pH within the acidic range resulted in a strong DHA inhibition, when compared to alkaline soils [39]. The pollution levels are frequently linked to the pH of contaminated sites, as microorganisms may not be able to transform PAHs under acidic or alkaline conditions. The extreme pH values that can be observed in some soils negatively influence the ability of microbial populations to degrade PAHs [46].

The aim of this experiment was to demonstrate the effect of six commercial formulations with effective microorganisms (EM) and one with a mixture of yeasts on the degradation in the soil of 13 substances belonging to PAHs. Furthermore, the analyses focused on shifts in the soil pH, the ORP, and the activity of dehydrogenases (DHA). The novelty of the approach used in this study lies in the analysis of biodegradation for the mixture of PAHs, instead of individual substances, and in the use of commercial preparations. These preparations promote the pro-environmental method of agricultural production – organic farming, and are easily accessible and safe for the environment and humans; therefore, they can be generally used. To this date, the influence of various microorganisms on the degradation of one or several PAHs has been studied. There are many different PAH compounds in the natural environment which never occur individually, but as a chemical mixture. Therefore, the research presented in this article, focusing on the EM influence on the mixture of 13 PAHs, reflects the conditions most similar to natural ones. The presented study also shows differences in the decomposition of individual PAHs by single microorganisms and by consortia of bacteria and yeasts. To our knowledge, no data and publications are available on the influence of tested EM formulations on the degradation of a PAHs mixture in the soil. The proposed research would be advantageous for the development of bioremediation of soils polluted with PAHs. The obtained results can significantly contribute to the development of such disciplines as environmental protection, biotechnology, agronomy, toxicology, and microbiology.

## 2 Materials and methods

Six commercial formulations containing EM and a mixture of formulations containing yeasts were used in the study. These are biological preparations and formulations for soil revitalization, having a potential to degrade persistent environmental pollutants. They are easily accessible and safe for the environment and humans; therefore, they can be generally used.

Table 1 provides the most important information about the formulations.

**Table 1:** Names and composition of EM formulations [47–52]

Formulation name (name of the manufacturer, country of origin)	Formulation composition
**Formulation 1** **–** **EmFarma Plus™** (ProBiotics, Poland)	Bacteria from the genus: *Bifidobacterium, Lactococcus, Lactobacillus, Bacillus, Rhodopseudomonas,* and *Streptococcus,* yeasts *S. cerevisiae,* revitalized water, rock salt, organic sugar cane molasses, and a mineral complex
**Formulation 2** **–** **Rewital PRO+** (BIOGEN, Poland)	Bacteria from the genus: *Streptomyces, Bacillus, Pseudomonas, Cellulomonas, Rhodococcus, Pseudonocardia, Arthrobacter,* and *Paenibacillus,* starter medium
**Formulation 3** **–** **BACILLUS VIP Probiotic Microorganisms** (AGROBIOS, Poland)	Eight bacteria strains from the *Bacillus* genus: *B. coagulans, B. amyloliquefaciens, B. laterosporus, B. licheniformis, B. mucilaginosus, B. megaterium, B. polymyxa,* and *B. pumilus,* revitalized water and organic sugar cane molasses
**Formulation 4** **–** **Myco Sin** ^**®**^ (Biocont, Poland)	Aluminum sulfate tetradecahydrate, inactive, ground, dried yeast – *S. cerevisiae*, and dry horsetail extract (*Equisetum arvense* L.)
**Formulation 5** **–** **Biopuls Fusion** ^**®**^ (Microlife, Poland)	Yeast strains *Y. lipolytica*
**Formulation 6** **–** **Biopuls Cinderella** (Microlife, Poland)	Yeast *D. hansenii* and its metabolites, bacteria from *Bacillus* genus
**Formulation 7**	Mix of yeast Formulations 4, 5, and 6

### 2.1 Reagents

Acetone, *n*-hexane of analytical grade, and petroleum ether for GC were obtained from Chempur, Poland. Salts used for extraction using the QuEChERS method included sodium chloride, magnesium sulfate, disodium citrate sesquihydrate, trisodium citrate (Chempur, Poland), together with sorbents used for clean-up – primary and secondary amines, PSA (Agilent, USA), and magnesium sulfate (Chempur, Poland). A certified mixture of standard solutions, EPA 525 PAHs Mix B, was obtained from Sigma-Aldrich, USA. Furthermore, methanol LC-MS (Honeywell, USA), 2,3,5-triphenyltetrazolium chloride – TTC (Sigma-Aldrich, USA) and 1,3,5-triphenyl tetrazolium formazan – TPF (Tokyo Chemical Industry, Japan) were used for the DHA analysis.

### 2.2 Soil samples preparation

In the experiment, a commercial universal soil recommended for the horticultural crop was used, containing high and low moor peat, sand, pine bark, dolomite, perlite, and mineral fertilizers. The soil had the salt content at the level of 0.5–1.0 g KCl/dm^3^ (the pH of 6.0–7.3) (PPUH Zielona Oaza I, Brzozów, Poland).

The studies were performed in the constant conditions of the ambient temperature of 21 ± 1°C and the soil humidity of about 70–74%.

Soil samples of 200 g were weighted into transparent 2 L polypropylene containers. About 40 mL of PAHs aqueous solution at 0.5 mg/kg was then added to each container. The samples were thoroughly mixed. On Day 4 of the experiment, PAHs content in each container was analyzed. These values were taken as initial and treated as 100% for calculating degradation on Day 35 of the experiment. Then, 40 mL of the EM formulations was added to each sample, as shown in the study plan presented in Table 2. The control samples, which contained only the studied PAHs, were spiked with water. All samples were mixed and analyzed in three replicates.

**Table 2:** Study plan and EM formulation doses

Sample number	Sample content	Formulation dose
1–3	Soil + PAHs	—
4–6	Soil + PAHs + Formulation 1	200 mL/L
7–9	Soil + PAHs + Formulation 2	10 mL/L
10–12	Soil + PAHs + Formulation 3	8 mL/L
13–15	Soil + PAHs + Formulation 4	50 g/L
16–18	Soil + PAHs + Formulation 5	100 mL/L
19–21	Soil + PAHs + Formulation 6	10 g/L
22–24	Soil + PAHs + Formulation 7	50 g/L, 100 mL/L, 10 g/L

The samples for analyses of PAHs were taken 4, 14, and 35 days after the PAHs application. The water content was measured using the weighing method, after drying at 105°C (S–40, Alpina, Poland). The pH and the ORP were determined using a digital meter ORP/pH AD14 (ADWA, Poland).

Figures S14 and S15 show shifts in pH values and the ORP in the soil samples on Days 4, 14, and 35 of the experiment.

### 2.3 Chromatographic analysis

Samples for PAHs analyses were prepared using a modification of the method described in PN–EN 15662: 2018-06. Foods of plant origin [53,54]. In brief, 5 g of the soil with 10 mL of water and 10 mL of the acetone: hexane mixture (1:4 v/v) were vortexed for 1 min (BenchMixerTM, Benchmark, USA). Next, extraction salts: 1 g of sodium chloride, 4 g of magnesium sulfate, 0.5 g of disodium citrate sesquihydrate, and 1 g of trisodium citrate were added. Then, samples were vortexed for 1 min and centrifuged at 3,500 rpm (5804R, Eppendorf, Hamburg, Germany) for 5 min. Then, 5 mL aliquots of the sample extract were poured into 15 mL polypropylene centrifuge tubes, containing sorbents for cleanup (150 mg of PSA and 900 mg of magnesium sulfate). The samples were vigorously shaken for 0.5 min and then centrifuged at 3,500 rpm for 5 min. PAHs in the soil extracts were analyzed with a 7890A gas chromatograph (Agilent Technologies, Palo Alto, CA, USA) coupled with a mass detector, model 7000 (GC-MS/MS QqQ). Software Mass Hunter, B.07.06, was used for data processing and data acquisition [54].

### 2.4 Assessment of DHA

DHA determination in the soil samples was conducted according to Casida et al., Tabatabai, and Wołejko et al. [55–57]. In brief, 4 mL of water and 1 mL of 3% aqueous solution of TTC were added to 6 g soil samples. Samples were then incubated in the dark at 37°C for 20 h. Next, 20 mL of methanol were added, vortexed for 1 min, centrifuged, and filtered. The DHA measurements were conducted at 485 nm with the spectrophotometer Cary 300 Bio (VARIAN, USA). The results were presented as micromoles of TPF/1 g of dry soil per 20 h.

### 2.5 Statistical analysis of results

Statistically significant differences between samples with and without biological formulations were established with the Student’s test (Excel Microsoft 365 program) for each individual sampling day. *p* values that are statistically significant, are presented in Figures S1, S2, S4–S8, S10, S12, S13, and S16 as *p* < 0.001 (***), *p* < 0.01 (**), and *p* < 0.05 (*).

## 3 Results and discussion

The effect that commercial formulations containing EM have on the degradation of 13 PAHs in the soil is described. Additionally, the analyses covered shifts in the soil pH, the ORP, and the DHA.

Microorganisms that are indigenous to a petroleum contaminated site were reported to be more effective in remediation of the environment after spills of oil and other pollutants, than the nonindigenous ones [58–62]. Many studies concern PAHs biodegradation by microorganisms isolated from different sites contaminated with crude oil. The tested organisms included bacteria belonging to genera *Nocardia, Pseudomonas, Gordonia, Micrococcus, Rhodococcus, Arthrobacter, Mycobacterium, Flavobacterium, Corynebacterium, Klebsiella, Alcaligenes,* and *Bacillus,* and fungi such as *Penicillium, Aspergillus, Trichoderma*, *Candida*, and *Fusarium* [27–31].

### 3.1 EM formulations influence on PAHs degradation

The study analyzed the effect of EM microorganisms from the following formulations: EmFarma Plus™, Rewital PRO+, BACILLUS VIP, Myco Sin^®^, Biopuls Fusion^®^, and Biopuls Cinderella, and a mix of preparations: Myco Sin^®^, Biopuls Fusion^®^, and Biopuls Cinderella, on the degradation of 13 PAHs in the soil. Below, we present a review of the literature on the PAHs degradation by microorganisms. Reports concerning the degradation of individual PAHs by microorganisms or by consortia of microorganism in the soil are available. However, to this date, there are no studies concerning the influence of microorganisms found in the studied formulations on the degradation of a mixture of 13 PAHs in the soil.

Table 3 shows a percentage degradation of the studied PAHs after EM formulations were used, on Day 35 of experiment, when compared to the control samples. For some of PAHs, in the case of Formulations 5, 6, and 7, the degradation was slightly lower than for control, but these differences were not statistically important at the end of the experiment on Day 35. This could be caused by a decrease in pH in soil samples after the application of preparations 5, 6, and 7, compared to the other formulations. According to Pawar [46], extremes in the pH, which can be observed in some soils, can negatively influence the ability of microbial populations to degrade PAHs.

**Table 3:** Percentage PAHs degradation (%) after formulations with EM were applied on Day 35 versus the initial concentrations

Analyzed substance	Control	Formulation 1	Formulation 2	Formulation 3	Formulation 4	Formulation 5	Formulation 6	Formulation 7
Acenaphthylene	82.6	91.9	92.7	91.8	91.3	90.7	89.2	87.5
Anthracene	90.3	94.5	93.9	95.1	92.2	92.3	91.4	92.9
Benzo[*a*]anthracene	85.2	90.7	90.4	90.8	88.9	89.6	90.3	88.2
Benzo[*a*]pyrene	77.5	87.3	85.1	83.6	85.2	85.3	82.0	86.2
Benzo[*b*]fluoranthene	79.0	88.4	86.4	84.9	86.6	86.2	78.5	83.8
Benzo[*k*]fluoranthene	75.1	86.8	85.1	83.3	84.9	84.5	75.5	81.5
Benzo[*ghi*]perylene	79.8	86.3	82.7	82.9	81.4	79.3	79.3	76.6
Chrysene	75.9	85.5	80.8	82.0	81.7	80.3	80.8	82.6
Dibenzo[*a,h*]anthracene	80.5	87.9	85.4	82.0	85.6	83.2	77.5	84.0
Fluorene	92.6	94.6	93.5	95.5	93.3	92.0	91.8	91.8
Indeno[1,2,3-*cd*] pyrene	70.0	85.1	83.7	85.6	85.3	87.0	84.4	83.3
Phenanthrene	85.3	90.8	87.8	88.6	87.8	83.6	83.1	83.8
Pyrene	84.2	91.8	90.1	90.7	89.3	88.9	85.9	87.3

Figures S1–S13 present PAHs decomposition on Day 4 (before application of EM formulation) and in the samples spiked with EM formulations on Days 14 and 35 of the experiment.

The results and the literature review of the 13 PAHs studied are presented below. No correlation was found between the number of rings and the partition coefficient LogP, and the rate of the tested PAHs degradation. The results were presented according to the molecular mass.

#### 3.1.1 Acenaphthylene

On Day 4 of the study, the acenaphthylene concentration ranged between 0.052 and 0.089 mg/kg. On Day 14 of the study, the acenaphthylene concentration was the highest in the control samples and amounted to 0.02 mg/kg, while it reached the lowest level in the samples with Formulations 4 and 5, amounting to 0.013 mg/kg. On the last, 35th day of the study, the acenaphthylene concentration was also the highest in the control samples (0.014 mg/kg), and it dropped to the minimum level in the samples treated with Formulations 4 and 5 (0.005 mg/kg) (Figure S1).

The use of all EM formulations accelerated the acenaphthylene degradation, when compared to the control. The achieved degradation was the lowest with Formulation 7 (yeast mix – 87.5%) and the highest (92.7%) with Formulation 2, containing bacteria from *Arthrobacter, Bacillus, Cellulomonas, Paenibacillus, Pseudonocardia, Streptomyces, Pseudomonas,* and *Rhodococcus* genera (it increased the rate of the acenaphthylene decomposition by 10.1% versus the control) (Table 3).

Rocha et al. analyzed the influence of *Pleurotus ostreatus* (the oyster fungus, edible mushroom) on the acenaphthylene degradation in the sandy soil. *P. ostreatus* degraded 57.7% of this compound in the soil enriched at the level of 30 mg/kg, and 65.8% of acenaphthylene when the soil was enriched at the level of 60 mg/kg, after the incubation period of 15 days [63].

Barnes et al. investigated the degradation of crude oil and associated PAHs using ten fungal cultures isolated from the aquatic environment: *Penicillium citrinum, Acremonium sclerotigenum, Aspergillus polyporicola, Aspergillus versicolor, Fusarium equiseti, Fusarium* sp., *Aspergillus* sp., *Aspergillus favus*, and *Aspergillus sydowii*. The studies were conducted in 20 mL of the mineral salt medium (MSM) containing 1% (w/v) crude oil as the sole source of carbon for isolates. The experimental flasks were incubated at 28°C with constant shaking at 80 rpm, for 23 days. Among the ten isolates studied, 100% of acenaphthylene was removed from six isolates [64].

#### 3.1.2 Fluorene

On Day 4, the fluorene concentration ranged from 0.031 to 0.072 mg/kg. On Day 14, the noted substance concentration was the highest in the control samples, amounting to 0.013 mg/kg, and the lowest in the samples treated with Formulations 4 and 5, of 0.008 mg/kg. On Day 35 of the experiment, fluorene was at the highest concentration, of 0.004 mg/kg, in the control samples and after application of Formulations 1 and 2, and at the lowest level, of 0.002 mg/kg, in the samples treated with Formulation 3 (Figure S10).

Fluorene proved to be a persistent substance. After the use of Formulations 5, 6, and 7, its degradation was lower versus the control (91.8–92.0%). The highest degradation was obtained for Formulation 3, at the level of 95.5% (Table 3).

Barnes et al. studied the degradation of the fluorene using ten fungal cultures, isolated from the aquatic environment: *P. citrinum, A. sclerotigenum, A. polyporicola, A. versicolor, F. equiseti, Fusarium* sp., *Aspergillus* sp., *A. favus*, and *A. sydowii.* Among the ten isolates studied, the degradation level ranged from 53.7 to 100% with *Aspergillus* sp., after the incubation period of 23 days [64].

Bankole et al. investigated the fluorene degradation efficiency of the marine derived filamentous fungus, *Mucor irregularis*, bpo1 strain. Optimization of the vital constituents of the MSM used in the study resulted in the fluorene degradation at the rate of 79.8%. The enhanced fluorene degradation efficiency (82.5%) was recorded when the optimized process variables were subjected to growth-linked validation experiments. Furthermore, the activity of enzymes, including laccase, manganese peroxidase, and lignin peroxidase, was demonstrated [65].

Nam et al. researched the influence of *Sphingobacterium* sp. KM-02, isolated from the soil polluted with PAHs near a mine-impacted area, on the fluorene degradation. During culturing of microorganisms with fluorene as the only source of carbon, decomposition of 78.4% of that PAH occurred within 120 h. Additionally, *Sphingobacterium* sp. KM-02 ability to biodegrade fluorene at a level of 100 mg/kg in the soil in the laboratory conditions was verified. During 20 days of the experiment, 65.6% of fluorene was decomposed [66].

Forty-seven fungal strains were isolated from the soil polluted with PAHs and the fluorene degradation rate was verified. The most productive of isolated strains was *Absidia cylindrospora*, and it was found that over 90% of fluorene was degraded within 288 h, while the process required 576 h when no microorganisms were present [67].

#### 3.1.3 Anthracene

Another studied substance was anthracene, and on Day 4, its concentration was in the range from 0.028 to 0.072 mg/kg. On Day 14, the noted substance concentration was the highest in the control samples, amounting to 0.017 mg/kg, while it was the lowest, of 0.008 mg/kg, in the samples treated with Formulation 5. On the last day of the study, anthracene also was at the highest concentration in the control samples, of 0.005 mg/kg, and at the minimum level of 0.002 mg/kg in the samples treated with Formulations 3, 6, and 7 (Figure S2).

Also in the case of anthracene, EM formulations accelerated its degradation, and in their presence, it ranged between 91.4 and 95.1%. The anthracene degradation was the highest following the treatment with Formulation 3, which contained eight bacteria strains from the *Bacillus* genus (Table 3).

The influence of microorganisms isolated from the soil long polluted with creosote oil on the anthracene degradation was studied by Smułek et al. The level of this compound degradation ranged from 30 to 70%. The highest degradation was achieved for *Pseudomonas mosselii* and *Pseudomonas mendocina* [68].

Krivobok et al. isolated from the soil 39 strains of *Micromycetes* fungi, described as effectively degrading PAHs. Nineteen of them degraded at least 50% of anthracene. The highest degradation was obtained for *Rhizopus arrhizus* – 95% and *Cryphonectria parasitica* – 96%. Furthermore, studies were also conducted on other fungal species able to degrade anthracene, including *Rhizoctonia solani* (86%), *Ceriporiopsis subvermispora* (88%), *Oxysporus* sp. (94%), *Cladosporium herbarum* (85%), *Drechslera spicifera* (79%), *Verticillium lecanii* (77%), *Coniothyrium sporulosum* (57%), and *Cunninghamella* spp. (78–87%) [69].

Two strains of bacteria able to degrade anthracene – *Ralstonia pickettii* JANC1A and *Thermomonas haemolytica* JANC2B, were isolated and identified. They were isolated using a method for enrichment of the contaminated soil in the mineral medium. The initial anthracene concentration amounted to 100 mg/kg. It was demonstrated that 50 and 75% of anthracene was degraded after 8 and 20 days, respectively [70].

Wu et al. investigated the anthracene degradation by fungi isolated from the environment of PAH-contaminated mangrove sediments in Ma Wan, Hong Kong. Anthracene at the concentration of 50 mg/L was added to the MSM for initial screening of PAH-degrading fungi, and finally two fungal species capable of using the studied PAH as the sole source of carbon were isolated and identified as *Fusarium solani* – MAS2 and MBS1 strains. Anthracene removal reached 40% of the added amount after 40 days of incubation in samples with MAS2 strain [71].

#### 3.1.4 Phenanthrene

On Day 4, the phenanthrene concentration ranged from 0.03 to 0.067 mg/kg. On Day 14, the highest substance level was 0.016 mg/kg and it was noted in the control samples, while its concentration was the lowest, of 0.009 mg/kg, in Formulations 4 and 5. On Day 35 of the study, phenanthrene reached the highest concentration of 0.008 mg/kg in the control samples, and the lowest, of 0.004 mg/kg, in the samples treated with Formulation 4 (Figure S12).

The use of Formulations 5, 6, and 7 slowed the phenanthrene degradation process versus the control level; however, these differences were not statistically significant for Day 35. The remaining formulations accelerated the degradation. The highest degradation, of 90.8%, was achieved with Formulation 1 (Table 3).

The phenanthrene biodegradation by *Bacillus. licheniformis* STK 01, *Bacillus subtilis* STK 02, and *Pseudomonas aeruginosa* STK 03, microorganisms that were isolated from an oil spill site, was studied. The phenanthrene concentration amounted to 50 mg/kg of the soil. After the experiment period of 60 days, 91.43, 84.83, and 83.97% of phenanthrene were decomposed by *B. licheniformis*, *B. subtilis*, and *P. aeruginosa*, respectively. When *B. licheniformis* and *B. subtilis* were used, 90.34% of phenanthrene was decomposed [72].

Nzila et al. isolated and characterized two bacterial strains able to degrade phenanthrene – *Pseudomonas citronellolis* PHC3Z1A and *Stenotrophomonas maltophilia* JPHC3Z2B. The initial phenanthrene concentration (*C*_0_ = 100 ppm) was degraded in 50% after 7 days and in 75% after 15 days [70].

A fungus, *Fusarium* sp., was isolated from soils polluted with crude oil (Liaohe Oil Field, China). The influence of that microorganism on the phenanthrene and pyrene degradation was studied. For both PAHs, four different initial concentrations, 10, 50, 100, and 200 mg/kg, were used. It was demonstrated that when higher initial concentrations were used, of 100 and 200 mg/kg, the phenanthrene degradation was higher, at the level of 83.7 and 70%, respectively (after 350 h), than when its initial levels were lower. For pyrene, biodegradation was the highest, of 74.6%, for the initial pyrene level of 100 mg/kg, and the lowest, of 32.1%, when it was at the level of 200 mg/kg [73].

Barnes et al. investigated the phenanthrene degradation using ten fungal cultures isolated from the aquatic environment: *P. citrinum, A. sclerotigenum, A. polyporicola, A. versicolor, F. equiseti, Fusarium* sp., *Aspergillus* sp., *A. favus,* and *A. sydowii.* Among the ten isolates studied, the degradation ranged from 22.1 to 100% after 23 days of incubation. The highest degradation level was achieved with *F. equiseti* [64].

The microbial degradation of the phenanthrene by screened fungi found in the natural environment was conducted, to select fungi for the phenanthrene bioremediation. The highest degradation level, of 72%, was obtained when *Trichoderma* sp. S019 was incubated for 30 days after 0.1 mM of phenanthrene were added to the liquid medium, while it reached 31% when 1 mM of the studied PAH was added [74].

#### 3.1.5 Pyrene

On Day 4 of the study, the highest pyrene concentration amounted to 0.05 mg/kg, and the lowest amounted to 0.019 mg/kg. On Day 14 of the study, pyrene reached its highest level of 0.011 mg/kg in the control samples, and the lowest level, of 0.006 mg/kg, in the samples treated with Formulation 5. On the last day of the study, pyrene was at the highest level, of 0.006 mg/kg, in the control samples, and at the lowest concentration of 0.003 mg/kg in the samples treated with Formulations 3, 4, 5, and 6 (Figure S13).

It was observed for pyrene that all studied EM formulations accelerated its degradation, which ranged between 85.9 and 91.8%. The degradation was the highest after treatment with Formulation 1, and the lowest for Formulation 6 (Table 3).

The studies on the influence of the immobilized (a hybrid carrier consisting of polyvinyl alcohol with sodium alginate and activated carbon) fungi, *Aspergillus niger*, *Trichoderma* sp., and *Fusarium* sp., on the pyrene degradation were conducted by Wang et al. The initial pyrene concentration was 100 mg/kg. After 240 h of incubation, the pyrene degradation amounted to 63% for *Trichoderma* sp., 49% for *A. niger*, and 69% for *Fusarium* sp. In the case of the synergistic effect of *A. niger* and *Fusarium* sp., the highest pyrene degradation of 81% was recorded [75].

A strain of the bacterium *Achromobacter xylosoxidans* PY4 was isolated from a polluted area (Jubail, Saudi Arabia) and characterized. It was demonstrated that this bacterium is able to metabolize and use pyrene as its only source of carbon. The PY4 strain was capable of decomposing 80% of pyrene at the initial level of 100 mg/L in 25 days [76].

Barnes et al. analyzed the pyrene degradation using ten fungal cultures, isolated from the aquatic environment, of *P. citrinum, A. sclerotigenum, A. polyporicola, A. versicolor, F. equiseti, Fusarium* sp., *Aspergillus* sp., *A. favus,* and *A. sydowii.* Among the ten isolates studied, the degradation ranged from 45.7 to 100% after 23 days of incubation. The maximum degradation level was observed when *F. equiseti* and *P. citrinum* were used [64].

The APC5 strain of *Coriolopsis byrsina,* white rot fungi, was isolated in the Surguja district of Chhattisgarh, India, and used in the studies on the pyrene biodegradation. The maximum degradation reached the level of 96.1%. *C. byrsina* produced a significant amount of ligninolytic enzyme in the mineral salt broth (MSB) containing pyrene [77].

Hadibarata et al. studied the pyrene degradation by *Candida* sp. S1, the yeast isolated from the tropical rain forest. Biodegradation was at the level of 35% after 15 days, but the percentage of pyrene decomposition increased up to 75% with 24 g/L of sodium chloride added, and decreased as the salinity increased. Under the acidic conditions, biodegradation increased up to 60% at the pH of 5. It was also found that higher glucose concentrations, exceeding 10 g/L, had no significant effect on the pyrene biodegradation, while agitation proved to have greater influence [31].

#### 3.1.6 Chrysene

On Day 4 of the study, the highest chrysene concentration amounted to 0.043 mg/kg, and the lowest amounted to 0.021 mg/kg. On Day 14, the substance was at the maximum concentration of 0.012 mg/kg in the samples treated with Formulation 7, and at the lowest, of 0.004 mg/kg, in the samples containing Formulation 5. On Day 35 of the study, chrysene level was the highest, of 0.008 mg/kg, in the control samples, and the lowest, of 0.004 mg/kg, in the samples treated with Formulations 4, 5, and 6 (Figure S8).

The use of all EM formulations accelerated the chrysene decomposition. The highest degradation, of 85.5%, was obtained for Formulation 1, while it was the lowest, of 80.3%, for Formulation 5 (yeast strains *Y. lipolytica*) (it accelerated the chrysene decomposition by 9.6% versus the control) (Table 3).

A fungus, *Polyporus* sp. S133, isolated from the soil polluted with crude oil, was used in the studies on the chrysene biodegradation in a culture without and with a synthetic surfactant (Tween 80). The maximum degradation intensity, of 86%, was achieved for the fungus incubated with 0.5% Tween 80 solution for 30 days. When the microorganism was incubated without that surfactant, the degradation was only at a level of 30% [78].

Similar studies were conducted in a liquid MSB. The maximum rate of the chrysene degradation, of 65%, was achieved when *Polyporus* sp. S133 was used with an addition of polypeptone as a source of vitamins, carbon, and amino acids for the microorganisms, when compared to the 24% degradation in the pure MSB medium [79].

Other research focused on the influence of a bacterial consortium on the chrysene degradation in the soil polluted with crude oil. The consortium consisting of *Bacillus cereus* and *Pseudomonas putida* 10 and 15% bacteria with ratios – 2:3, 1:1, 3:2 was added into a slurry bioreactor. The initial chrysene concentration amounted to 24.48 ng/µL. After 49 days of the experiment, the chrysene degradation was at the level 67.01, 69.1, and 64.54%, in the case of the 10% bacterial consortium, and of 89.39, 93.58, and 91.73%, for the 15% bacterial consortium [80].

In yet another study, Vaidya et al. developed a bacterial consortium consisting of *Rhodococcus* sp., ASDC1; *Bacillus* sp. ASDC2; and *Burkholderia* sp. to study the chrysene degradation. Chrysene was utilized by the consortium as a sole source of carbon and energy, with the maximum degradation rate of 1.5 mg/L/day and the maximum growth rate of 0.125/h, under optimized conditions of the pH of 7.0, the temperature of 37°C under aeration of 150 rpm, on gyrating shaking. The maximum degradation of 96% was obtained in the polluted, non-sterile soil sediment [81].

#### 3.1.7 Benzo[*a*]anthracene

On Day 4, the benzo[*a*]anthracene level in the soil samples was between 0.016 and 0.041 mg/kg. On Day 14 of the experiment, the maximum concentration was noted in the control samples and following treatment with Formulation 1, amounting to 0.008 mg/kg, while it was the lowest, of 0.004 mg/kg, after treatment with Formulations 3, 4, and 5. On the last day of the study, the benzo[*a*]anthracene level was the highest in the control samples and amounted to 0.005 mg/kg, while it was the lowest after treatment with Formulations 4, 5, and 6, and amounted to 0.002 mg/kg (Figure S3).

The benzo[*a*]anthracene decomposition was accelerated by the use of all formulations. It was the highest, of 90.8%, after treatment with Formulation 3, and the lowest, of 88.2%, with Formulation 7 (Table 3).

The potential of benzo[*a*]anthracene biodegradation by *Panebacillus* sp. strain HD1PAH, a new strain isolated from the soil polluted with crude oil, was studied by Deka and Lahkar. The study was conducted in laboratory conditions for 144 h, and samples were collected seven times. Benzo[*a*]anthracene was the only source of carbon, at the level of 10 mg/L in the MSM. The maximum degradation of that PAH by the *Panebacillus* sp. strain HD1PAH was 82.01% during 144 h of incubation [82].

Rocha et al. also analyzed the *P. ostreatus* influence on the benzo[*a*]anthracene degradation in the soil. *P. ostreatus* degraded about 90% of this compound in the soil enriched at the level of 30 mg/kg, and 80% of benzo[*a*]anthracene when the soil was enriched at the level of 60 mg/kg after a period of incubation of 15 days [63].

Barnes et al. analyzed the degradation of benzo[*a*]anthracene using ten fungal cultures, isolated from the aquatic environment: *P. citrinum, A. sclerotigenum, A. polyporicola, A.versicolor, F. equiseti, Fusarium* sp., *Aspergillus* sp., *A. favus,* and *A. sydowii.* Among the ten isolates studied, nine isolates achieved a 100% removal of benzo[*a*]anthracene after incubation of 23 days [64].

Wu et al. studied the degradation of benzo[*a*]anthracene by fungi isolated from the environment of PAH-contaminated mangrove sediments in Ma Wan, Hong Kong. Benzo[*a*]anthracene at the concentration of 20 mg/L was added to the MSM for initial screening of PAH-degrading fungi; and finally, two fungal species capable of using the studied PAH as the sole source of carbon were isolated and identified as *F. solani* – MAS2 and MBS1 strains. Benzo[*a*]anthracene removal reached 60% of the added amount after 40 days of incubation in the samples containing the MBS1 strain [71].

#### 3.1.8 Benzo[*a*]pyrene

On Day 4 of the study, the benzo[*a*]pyrene level was the highest of 0.039 mg/kg, and the lowest of 0.015 mg/kg. On Day 14 of the study, the compound concentration was the highest in the control samples and those with Formulation 1 (0.01 mg/kg), and the lowest in the samples spiked with Formulation 5 (0.004 mg/kg). On the last day of the study, the benzo[*a*]pyrene concentration was the highest, of 0.007 mg/kg, in the control samples, while it is at the minimum level of 0.003 mg/kg in the samples treated with Formulations 4, 5, 6, and 7 (Figure S4).

The use of all EM formulations accelerated the degradation of benzo[*a*]pyrene, when compared to the control, and it ranged between 82.0 and 87.3%. Formulation 1, containing bacteria from *Bifidobacterium, Bacillus, Lactococcus, Lactobacillus, Rhodopseudomonas,* and *Streptococcus* genera, and yeasts *S. cerevisiae*, most strongly accelerated the decomposition of this substance (it accelerated the benzo[*a*]pyrene degradation by 9.8% versus the control) (Table 3).

Su et al. studied the influence of two microorganisms, *Bacillus* sp. SB02 and *Mucor* sp. SF06, on the benzo[*a*]pyrene degradation in the soil. Both SF06 and SB02 were isolated from the soil collected from the Shenfu Irrigation Area, China. The researchers studied and compared characteristics of the benzo[*a*]pyrene degradation, by free and by co-immobilized microorganisms. The level of the benzo[*a*]pyrene degradation was verified five times, on Days 7, 14, 21, 28, and 42. A higher degradation of that compound was achieved for microorganisms in the co-immobilized system. The highest degradation level was 79.6% for free microorganisms, and 95.3% for co-immobilized microorganisms [83].

Su et al. conducted similar studies on the benzo[*a*]pyrene degradation in the soil by the fungus, *Mucor* sp., free and immobilized. This microorganism was isolated from the soil contaminated with PAHs from the Shenfu Irrigation Area. The initial concentration of the studied PAH was 50 mg/kg. The degradation rate was higher for the immobilized fungi. The maximum degradation amounted to 68% for the immobilized fungi and 52% for free microorganisms on Day 42 of the experiment [84].

The influence of two bacteria, *Bacillus flexus* S1I26 and *Paenibacillus* sp. S1I8, isolated from the soil polluted with crude oil was studied. It was demonstrated that both isolates were able to secrete biosurfactant that increased benzo[*a*]pyrene solubility. On Day 21 of the experiment, *B. flexus* S1I26 degraded 70.7% of benzo[*a*]pyrene, while *Paenibacillus* sp. S1I8 achieved a higher degradation level, amounting to 76.8%. The results suggest that isolates producing biosurfactants can be a potential tool for PAHs biodegradation in the soil and they can be used to bioremediate sites polluted with those compounds [85].

The studies concerning the influence of fungi, *Trichoderma* sp., *Fusarium* sp., and *A. niger*, on the benzo[*a*]pyrene degradation were conducted. A hybrid carrier consisting of polyvinyl alcohol, sodium alginate, and activated carbon was used to immobilize the microorganisms. The initial concentration of benzo[*a*]pyrene was 100 mg/kg. After 240 h of incubation, the benzo[*a*]pyrene degradation amounted to 34% for *Trichoderma* sp., 29% for *A. niger*, and 37% for *Fusarium* sp. The degradation levels significantly exceeded those obtained for free fungi. Additionally, the synergistic effect of those microorganisms was verified, and for benzo[*a*]pyrene the highest degradation was obtained with *A. niger* and *Fusarium* sp., amounting to 43% [75].

Mandal et al. analyzed the benzo[*a*]pyrene degradation by a yeast consortium including *Rhodotorula* sp. NS01, *H. opuntiae* NS02, *D. hansenii* NS03, and *H. valbyensis* NS04, isolated from the contaminated soil. The maximum degradation amounted to 76% after 6 days in the aqueous medium under optimized conditions of pH 7.0, temperature of 30°C, shaking speed of 130 rpm, an inoculum dose of 3% (w/v), and the initial benzo[*a*]pyrene concentration of 50 mg/L [35].

#### 3.1.9 Benzo[*b*]fluoranthene

On Day 4, the benzo[*b*]fluoranthene concentration was in the range from 0.013 to 0.039 mg/kg. On Day 14 of the study, the benzo[*b*]fluoranthene concentration was the highest in the control samples and amounted to 0.01 mg/kg, while it was the lowest in those with Formulation 5, amounting to 0.004 mg/kg. On the last day of the study, the studied substance was at the highest level, of 0.006 mg/kg, in the control samples, and at the minimum level of 0.003 mg/kg in the samples treated with Formulations 3, 4, 5, 6, and 7 (Figure S5).

The benzo[*b*]fluoranthene degradation was higher following treatment with EM formulations, excluding Formulation 6 (the yeast *D. hansenii* and its metabolites, bacteria from *Bacillus* genus), for which the degradation was similar to the control, at a level of 78.5%. The highest degradation, of 88.4%, was achieved for Formulation 1 (it accelerated the benzo[*b*]fluoranthene decomposition by 9.4% versus the control) (Table 3).

Kumari et al. demonstrated the ability of *Ochrobactrum anthropi, S. maltophilia, P. mendocina, P. aeruginosa,* and *Microbacterium esteraromaticum* to biodegrade several PAHs, including benzo[*b*]fluoranthene, during the bacteria exposure to crude oil. The benzo[*b*]fluoranthene concentration in the sample of oil collected from the Digboi refinery in India amounted to 6.5 mg/L. The experiments were conducted in laboratory conditions. The samples were tested on Days 15, 30, and 45. The degradation was the lowest, at the level of 34.8%, for *P. mendocina*, and the highest, of 61.2%, for *P. aeruginosa*. However, the consortium of the described bacteria achieved the enhanced benzo(b)fluoranthene degradation of 72.8% [86].

Treviño-Trejo et al. isolated and selected bacteria able to degrade benzo[*b*]fluoranthene. The isolates ability to tolerate concentrations of 50 and 75 mg/L of the studied PAH in the liquid medium was evaluated. The selected isolates were identified as belonging to *Bacillus, Gordonia, Pseudomonas, Rhodococcus, Ochrobactrum,* and *Amycolatopsis* genera. All isolates tolerated and grew at the benzo[*b*]fluoranthene concentrations tested. The most prominent was *Amycolatopsis* sp. Ver12, which removed 47% of benzo[*b*]fluoranthene; furthermore, with the addition of the yeast extract, it removed 59% of the studied compound [87].

#### 3.1.10 Benzo[*k*]fluoranthene

On Day 4 of the study, the highest benzo[*k*]fluoranthene concentration amounted to 0.043 mg/kg, and the lowest amounted to 0.013 mg/kg. On Day 14 of the study, the concentration was the at the highest level, of 0.011 mg/kg, for the control samples and the samples treated with Formulation 1, and the lowest, of 0.004 mg/kg, following treatment with Formulation 5. On the last, 35th day of the study, the studied substance was at the highest level, of 0.008 mg/kg, in the control samples, and at the lowest level, of 0.003 mg/kg, in the samples treated with Formulations 4, 5, 6, and 7 (Figure S6).

The use of all EM formulations accelerated the benzo[*k*]fluoranthene decomposition. The degradation was the lowest for Formulation 6 (75.5%), and the highest for Formulation 1 (86.6%) (it accelerated the benzo[*k*]fluoranthene decomposition by 11.7% versus the control) (Table 3).

The results for benzo[*b*]fluoranthene and benzo[*k*]fluoranthene are very similar, due to the similar structure of these two PAHs.

Arulazhagan et al. studied the benzo(k)fluoranthene degradation by *S. maltophilia* strain AJH1, bacteria isolated from soil samples collected from different areas owned by the Saudi Arabian Mining Company. Strain AJH1 was able to degrade the benzo(k)fluoranthene at the concentration of 10 mg/L in the acidophilic MSM at the pH of 2. The maximum degradation level was 79% after 11 days [88].

Maeda et al. reported the studies on the benzo(k)fluoranthene degradation by the soil bacterial isolates *Sphingobium* sp. strain KK22. Benzo(k)fluoranthene concentration was reduced by 70% in 20 days of the experiment [89].

#### 3.1.11 Dibenzo[*a,h*]anthracene

On Day 4 of the study, the highest dibenzo[*a*,*h*]anthracene concentration amounted to 0.041 mg/kg, and the lowest amounted to 0.014 mg/kg. On Day 14 of the experiment, the highest dibenzo[*a*,*h*]anthracene concentration, of 0.013 mg/kg, was found after treatment with Formulation 1. The lowest level, of 0.006 mg/kg, was noted in the samples treated with Formulations 5, 6, and 7. On Day 35 of the study, the substance was at its highest level, of 0.006 mg/kg, in the control samples, while it was at the lowest concentration, of 0.003 mg/kg, in the samples treated with Formulations 4, 5, 6, and 7 (Figure S9).

The use of EM formulations accelerated the dibenzo[*a*,*h*]anthracene degradation when compared to the control, except for Formulation 6 (77.5%). The highest degradation, of 87.9%, was achieved with Formulation 1 (Table 3).

The influence of eight fungal species *A. praecox, Agrocybe dura, Kuehneromyces mutabilis, Hypholoma capnoides, S. rugosoannulata, Stropharia coronilla, S. cubensis,* and *S. hornemannii,* on the dibenzo[*a*,*h*]anthracene degradation was studied by Steffen et al. The highest degradation of that compound, of 84%, was obtained for *S. coronilla* [90].

Dibenzo(a,h)anthracene biodegradation by indigenous strains of aerobic heterotrophic bacteria and cyanobacteria isolated from the Bodo creek, a moderately salty aquatic site polluted with crude oil, was monitored. Dibenzo(*a*,*h*)anthracene levels were reduced to 0 mg/L in all treatment options on Day 56 [91].

#### 3.1.12 Benzo[*ghi*]perylene

Benzo[*ghi*]perylene was yet another studied substance. On Day 4, this substance was at the highest concentration of 0.052 mg/kg, and at the lowest level of 0.020 mg/kg. On Day 14 of the study, the benzo[*ghi*]perylene concentration was the highest in the control samples and amounted to 0.019 mg/kg, while it was the lowest in the samples spiked with Formulations 5 and 7, amounting to 0.011 mg/kg. On Day 35, the concentration was the highest, of 0.008 mg/kg, in the control samples, and the lowest, of 0.004 mg/kg, in the samples treated with Formulation 6 (Figure S7).

After treatment with Formulations 5 and 6, the benzo[*ghi*]perylene degradation was comparable to the control (ca. 79%), and for Formulation 7 the degradation was lower, of 76.6%. The highest degradation was obtained for Formulation 1, of 86.3% (Table 3).

Studies were conducted on the benzo[*ghi*]perylene biodegradation by *B. licheniformis* STK 01, *B. subtilis* STK 02, and *P. aeruginosa* STK 03, which were isolated from wood chips, coal tar, and an oil spill site. In the experiment, mono-septic cultures or co- and augmented cultures were used. The benzo[*ghi*]perylene concentration was 25 mg/kg of the soil. After 60 days of the experiment, 52.73, 40.50 and 58.42% of benzo[*ghi*]perylene were biodegraded by *B. licheniformis*, *B. subtilis*, and *P. aeruginosa*, respectively. The highest rate of the benzo[*ghi*]perylene degradation, of 60.76%, was reached with *B. licheniformis* and *B. subtilis* [72].

Mandal et al. studied the benzo[*ghi*]perylene degradation by the yeast consortium YC04, consisting of three isolates – *Rhodotorula* sp. NS01, *D. hansenii* NS03, and *H. valbyensis* NS04 in the MSM. The YC04 efficiency in benzo[*ghi*]perylene remediation was tested in a presence of ZnO nanoparticles and produced a biosurfactant in the growth medium. The maximum benzo[*ghi*]perylene biodegradation was found to be 63.83% at central values of all factors, a pH of 7.0, at a temperature of 30°C, and at a shaking speed of 130 rpm in the presence of 2 g/L of ZnO nanoparticles, using 3% inoculum doses of the yeast consortium YC04 after 6 days of incubation [34].

#### 3.1.13 Indeno[1,2,3-*cd*] pyrene

On Day 4, the highest determined indeno[1,2,3-*cd*] pyrene level was 0.035 mg/kg, while the lowest concentration amounted to 0.015 mg/kg. On Day 14, the highest level of the studied substance was 0.031 mg/kg in the control samples, while the lowest concentration was found in the samples treated with Formulation 7 (0.014 mg/kg). On the last day of the experiment, the highest indeno[1,2,3-*cd*]pyrene level of 0.008 mg/kg was found in the control samples, while its level was the lowest (0.002 mg/kg) following application of Formulation 6 (Figure S11).

The use of EM formulations significantly accelerated the indeno[1,2,3-*cd*]pyrene degradation versus the control. The highest degradation, of 87%, was obtained for Formulation 5, while it was the lowest, of 83.3%, for Formulation 7 (it accelerated the indeno[1,2,3-*cd*]pyrene decomposition by 17% versus the control) (Table 3).

Two bacterial strains, *Acinetobacter baumannii* INP1 and *Pseudomonas taiwanensis* PYR1, were isolated from Liaohe, China, contaminated with PAHs. These microorganisms were able to degrade 55.3% of indeno[1,2,3-*cd*]pyrene after a period of 30 days. These strains’ ability to degrade pyrene was also studied, and it was found that they degraded 58.2% of pyrene [92].

Barnes et al. analyzed the degradation of indeno[1,2,3-*cd*]pyrene using ten fungal cultures, isolated from the aquatic environment – *P. citrinum, A. sclerotigenum, A. polyporicola, A.versicolor, F. equiseti, Fusarium* sp., *Aspergillus* sp., *A. favus,* and *A. sydowii.* All tested isolates degraded almost 100% of indeno[1,2,3-*cd*]pyrene [64].

### 3.2 Soil parameters: pH, the ORP, and DHA

On Day 1, the soil pH was 5.1, and on Day 4, after PAHs application, the pH of all soil samples was about 5.3. On Day 14, the pH of all studied samples increased, and this increase was the highest for the soil containing bacterial preparations. On Day 35, the pH decreased slightly or remained at the same level. On Days 14 and 35, in the samples with preparation 3 added, the soil pH value was the highest, of 5.9 and 5.6, respectively. The highest PAHs degradation was obtained for these samples. In acidic samples, the tested PAHs were more persistent (Figure S14). According to Pawar [46], the pollutants are frequently linked with the pH of contaminated sites, and microorganisms may not be able to transform PAHs under the acidic or alkaline conditions. Most heterotrophic bacteria and fungi favor a nearly neutral pH, with fungi being more tolerant of acidic conditions. Extremes in the pH, which can be observed in some soils, can negatively influence the ability of microbial populations to degrade PAHs.

On Day 14, following application of EM formulations, the ORP increased in all studied samples, when compared to the control samples, and was in a range from 295 (the sample containing Formulation 1) to 351 (the sample with Formulation 6). On Day 35 of the study, the ORP increased again and reached the highest value of 379 for the sample containing Formulation 7. On Day 35, ORP increased by almost 100, when compared to Day 1. On each day of testing, ORP for all samples with microorganisms was significantly higher versus the control samples (Figure S15). The ORP plays a crucial role in regulation of the microbial activity, and affects the soil enzymatic activity, which has an impact on the PAHs degradation [43].

The initial DHA value in the control samples was 26.5 µM TPF/g DM soil 20 h. The changes in the DHA are presented in Figure S16. The use of PAHs resulted in the DHA decrease in all samples on Day 14 versus Day 1. According to Karaca et al., Wolińska and Stępniewska, and Järvan et al. [39–42], it is well known that pesticides, PAHs, and other persistent soil pollutants have inhibiting effects on the DHA. On Day 14, DHA in the samples with biological preparations was higher than in controls, except for Formulations 2 and 3. On Day 35, for Formulation 3 (which had the best PAHs bioremediation results), DHA increased from 12.8 µM TPF/g DM soil 20 h to 24.4 µM TPF/g DM soil 20 h after decomposition of these pollutants. The addition of the biological formulations significantly changes the enzymatic activity of the soil, and this was still visible on Day 35 [57].

## 4 Conclusions

Formulation 1, containing bacteria from *Bacillus, Bifidobacterium, Lactococcus, Lactobacillus, Rhodopseudomonas,* and *Streptococcus* genera, and yeasts *S. cerevisiae* was demonstrated to have the highest effectiveness in the biodegradation of the studied PAHs. When this formulation was used, eight PAHs: benzo[*a*]pyrene, benzo[*k*]fluoranthene, benzo[*b*]fluoranthene, benzo[*ghi*]perylene, chrysene, dibenzo[*a*,*h*]anthracene, phenanthrene, and pyrene were degraded with the highest intensity. The biological formulations containing bacteria proved to be more effective than yeast formulations. The presented studies show differences in the PAHs degradation by commercially available individual microorganisms and consortia of bacteria and yeasts. Commercial preparations are easily accessible and safe for the environment and humans; therefore they can be generally used. The proposed research would be advantageous for the development of bioremediation of soils contaminated with PAHs. The studies on applications of biological preparations should be continued, because other parameters such as the soil type or environmental conditions could also influence the PAHs degradation.

## Supplementary Material

Supplementary Figure
