# Supplementary material for: Degradation of a mixture of 13 polycyclic aromatic hydrocarbons by commercial effective microorganisms
Source: Open Life Sci. 2024 Feb 5;19(1):20220831. doi: 10.1515/biol-2022-0831 (PMC10898624; doi:10.1515/biol-2022-0831)
Supplement: Supplementary Figure [file biol-2022-0831-sm.pdf]

Supplementary material

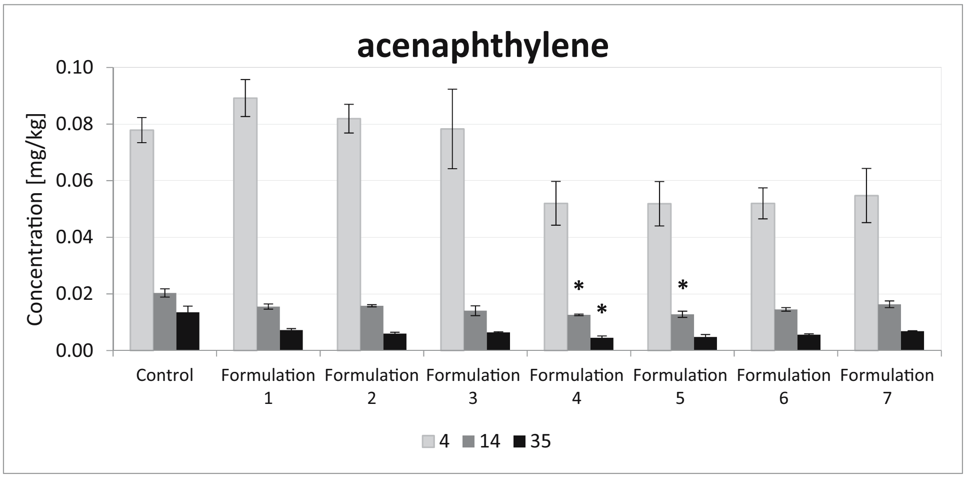

Figure S1: Acenaphthylene concentrations on Day 4 of experiment (before application of EM), and on Days 14 and 35 of experiment (after application of EM). Statistically significant *p* value is shown as $p < 0.05$ (*).

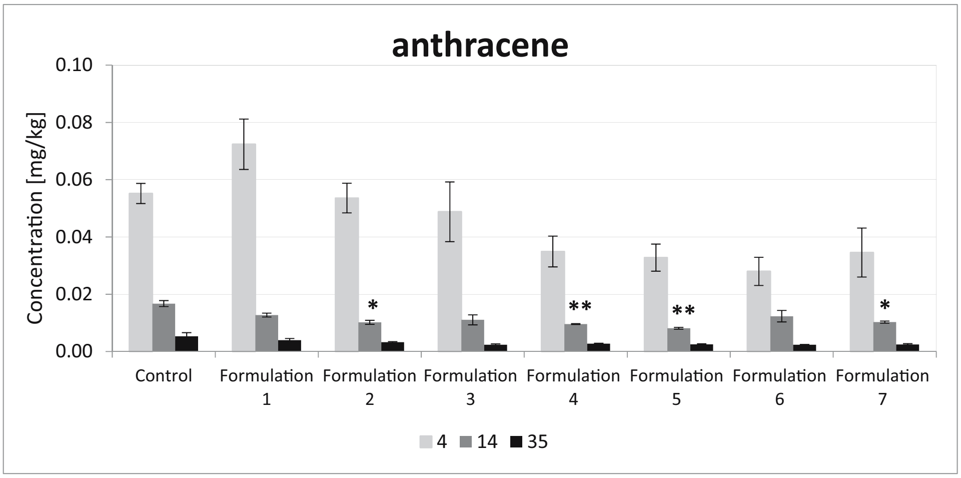

Figure S2: Anthracene concentrations on Day 4 of experiment (before application of EM), and on Days 14 and 35 of experiment (after application of EM). Statistically significant *p* values are shown as $p < 0.01$ (**) and $p < 0.05$ (*).

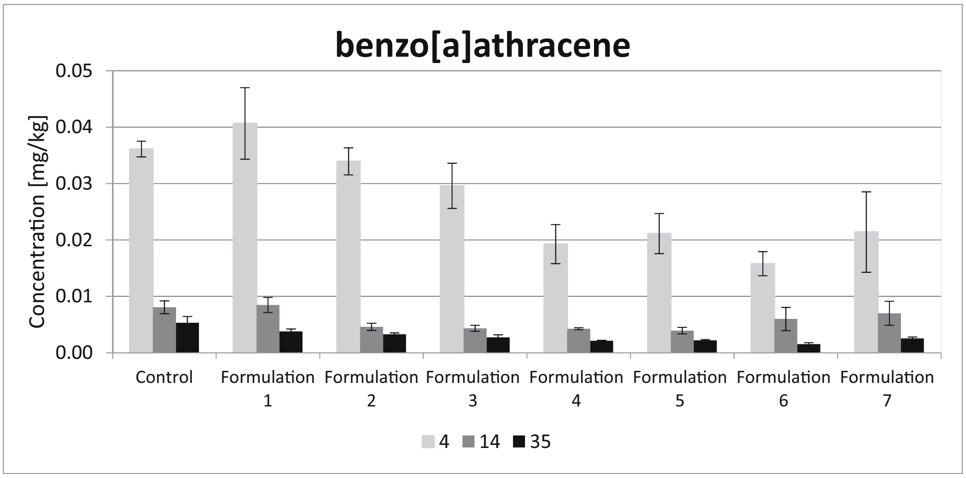

Figure S3: Benzo[a]anthracene concentrations on Day 4 of experiment (before application of EM), and on Days 14 and 35 of experiment (after application of EM). No statistically significant differences were observed.

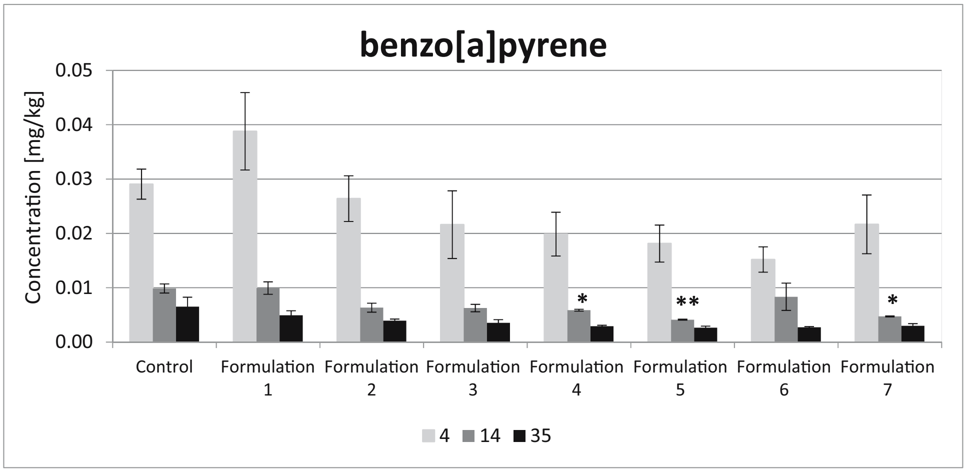

Figure S4: Benzo[a]pyrene concentrations on Day 4 of experiment (before application of EM), and on Days 14 and 35 of experiment (after application of EM). Statistically significant *p* values are shown as $p < 0.01$ (**) and $p < 0.05$ (*).

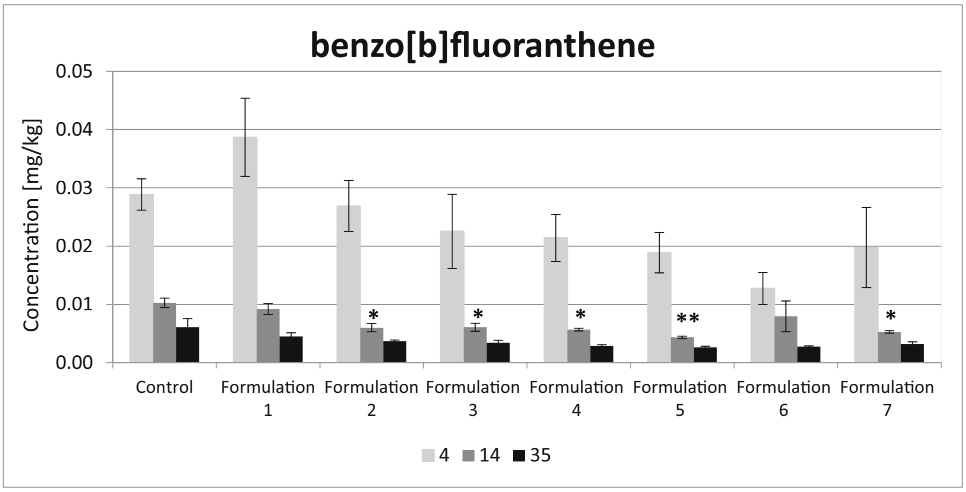

Figure S5: Benzo[b]fluoranthene concentrations on Day 4 of experiment (before application of EM), and on Days 14 and 35 of experiment (after application of EM). Statistically significant *p* values are shown as $p < 0.01$ (**) and $p < 0.05$ (*).

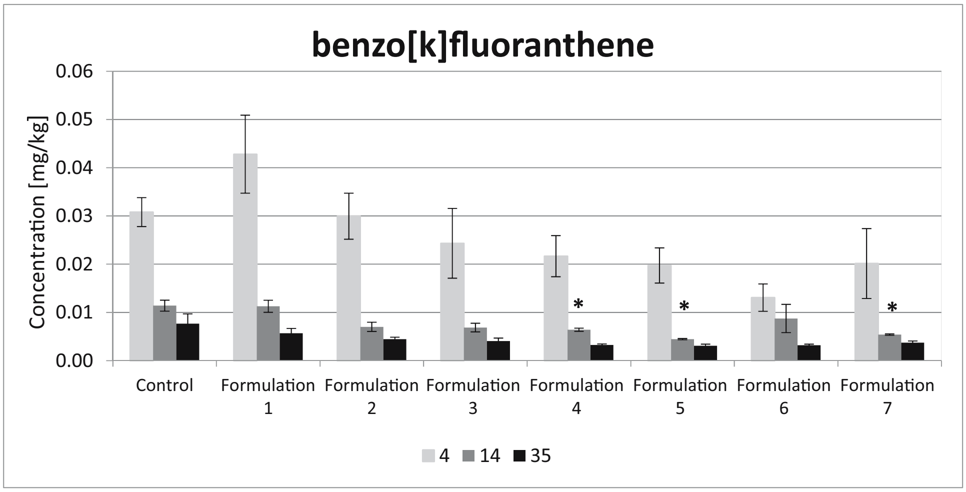

Figure S6: Benzo[k]fluoranthene concentrations on Day 4 of experiment (before application of EM), and on Days 14 and 35 of experiment (after application of EM). Statistically significant *p* value is shown as $p < 0.05$ (*).

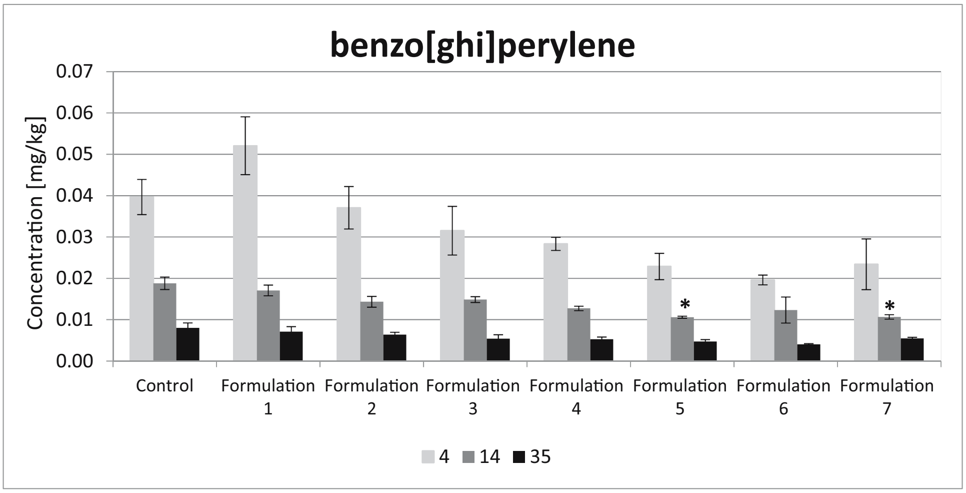

Figure S7: Benzo[ghi]perylene concentrations on Day 4 of experiment (before application of EM), and on Days 14 and 35 of experiment (after application of EM). Statistically significant *p* value is shown as $p < 0.05$ (*).

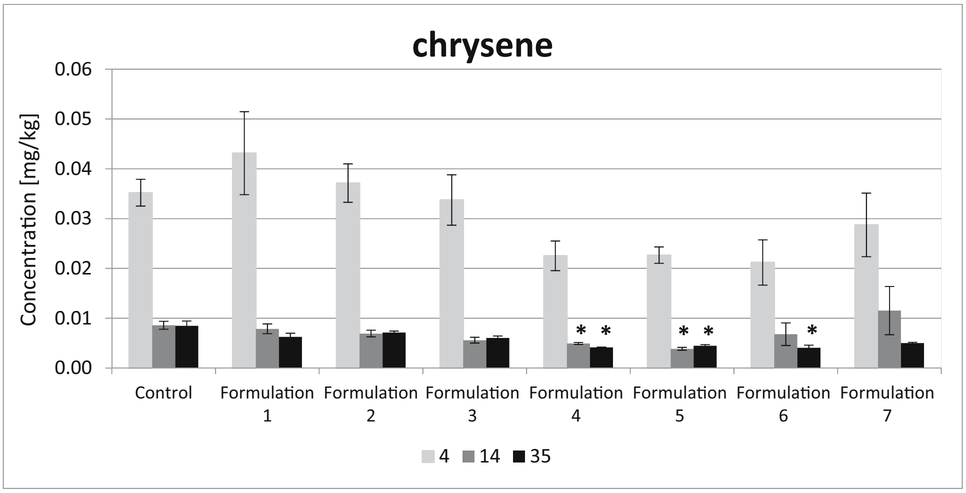

Figure S8: Chrysene concentrations on Day 4 of experiment (before application of EM), and on Days 14 and 35 of experiment (after application of EM). Statistically significant *p* value is shown as $p < 0.05$ (*).

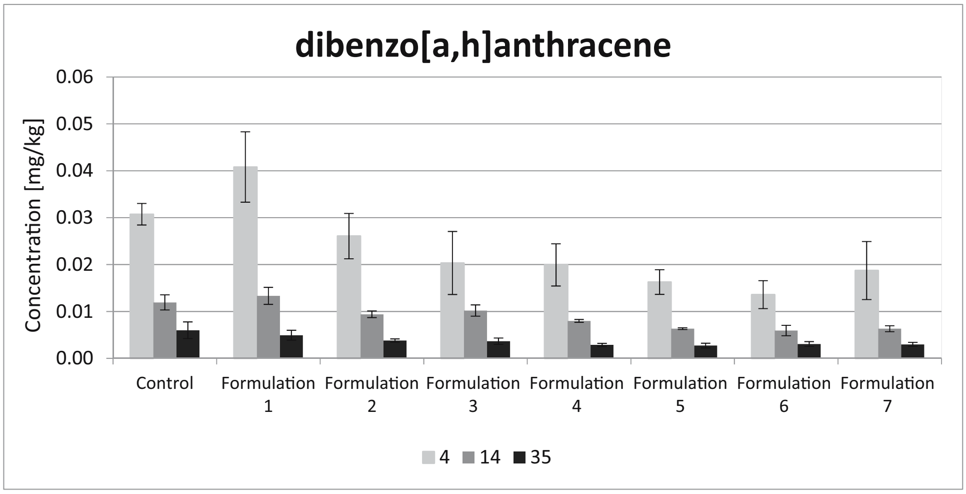

Figure S9: Dibenzo[a,h]anthracene concentrations on Day 4 of experiment (before application of EM), and on Days 14 and 35 of experiment (after application of EM). No statistically significant differences were observed.

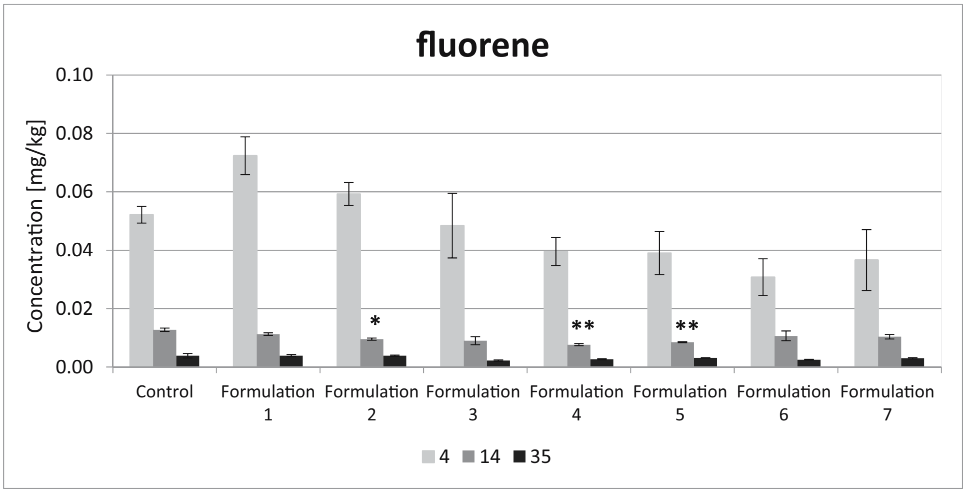

Figure S10: Fluorene concentrations on Day 4 of experiment (before application of EM), and on Days 14 and 35 of experiment (after application of EM). Statistically significant *p* values are shown as $p < 0.01$ (**) and $p < 0.05$ (*).

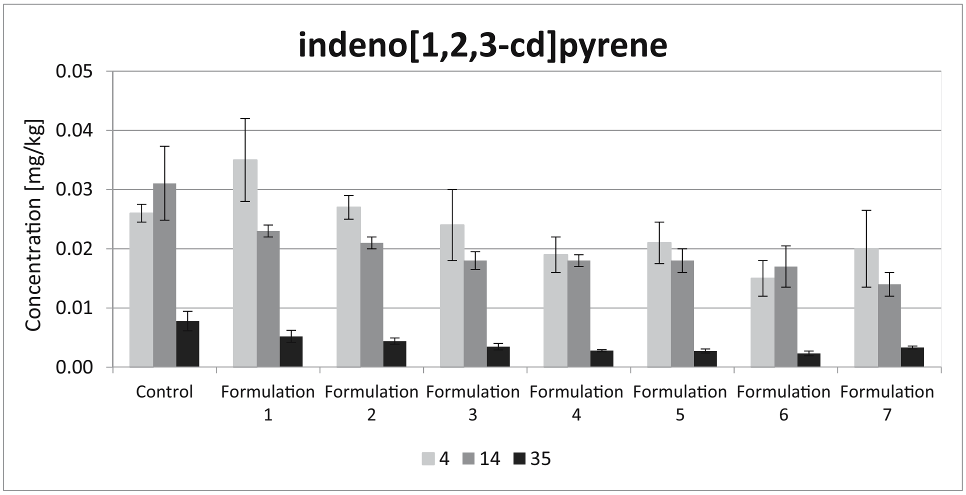

Figure S11: Indeno[1,2,3-cd]pyrene concentrations on Day 4 of experiment (before application of EM), and on Days 14 and 35 of experiment (after application of EM). No statistically significant differences were observed.

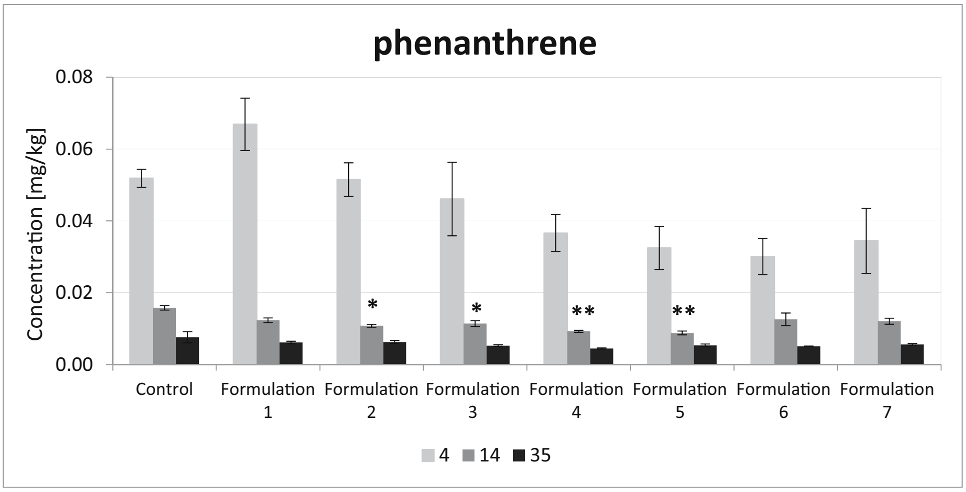

Figure S12: Phenanthrene concentrations on Day 4 of experiment (before application of EM), and on Days 14 and 35 of experiment (after application of EM). Statistically significant *p* values are shown as $p < 0.01$ (**) and $p < 0.05$ (*).

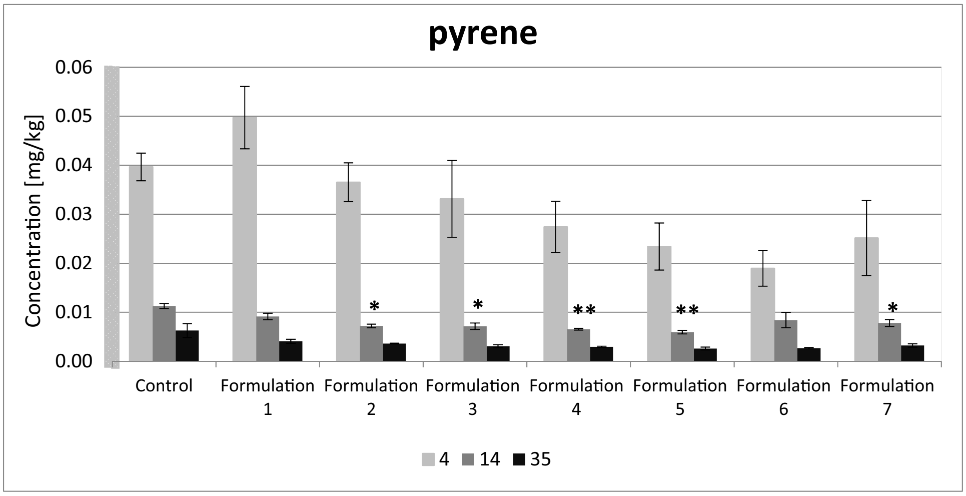

Figure S13: Pyrene concentrations on Day 4 of experiment (before application of EM), and on Days 14 and 35 of experiment (after application of EM). Statistically significant *p* values are shown as $p < 0.01$ (**) and $p < 0.05$ (*).

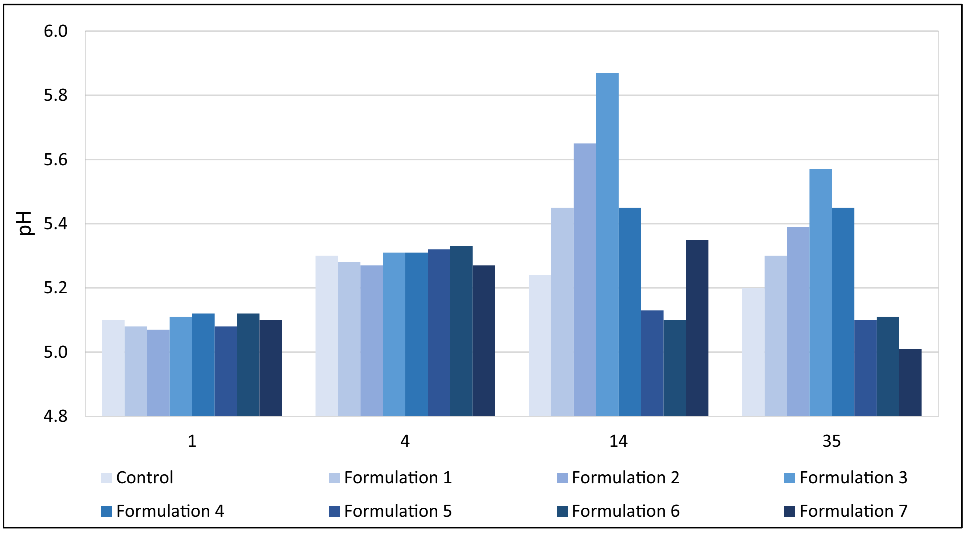

Figure S14: Shifts in the soil pH levels with PAHs and following treatments with EM formulations on successive days of the study, on Day 4 of experiment (before application of EM), and on Days 14 and 35 of experiment (after application of EM). ORP — oxidation-reduction potential.

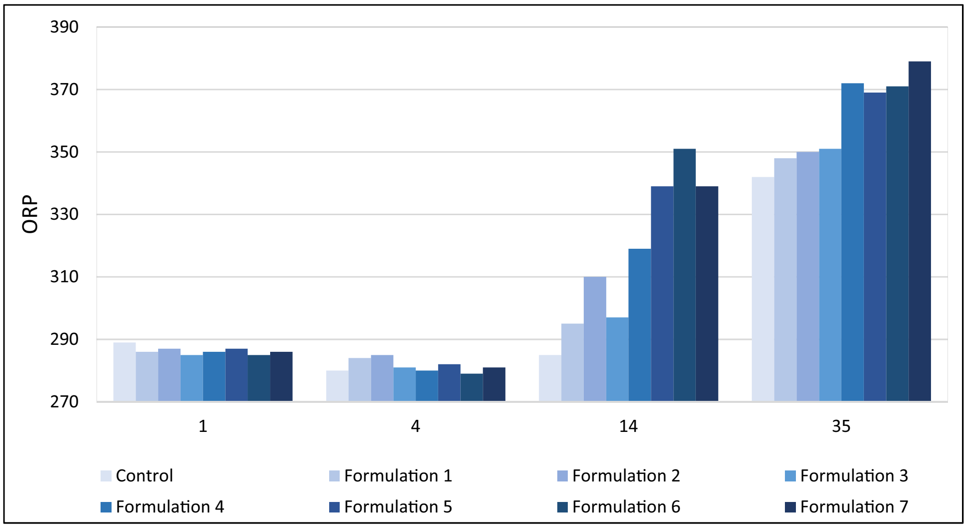

Figure S15: Shifts in the soil oxidation-reduction potential with PAHs and after treatment with EM formulations over time, on Day 4 of experiment (before application of EM), and on Days 14 and 35 of experiment (after application of EM).

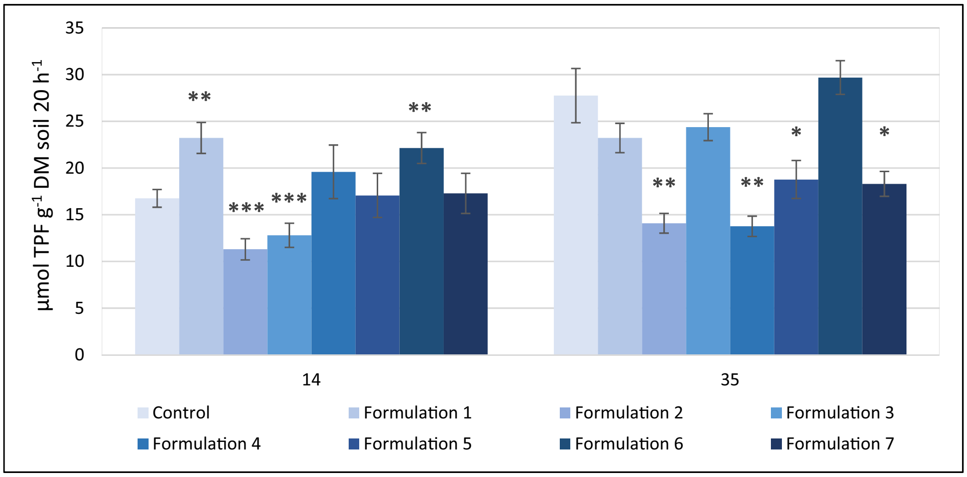

Figure S16: Shifts in the soil DHA with PAHs and after treatment with EM formulations over time. Statistically significant *p* values are shown as $p < 0.05$ (*), $p < 0.01$ (**) and $p < 0.001$ (***). Initial value of DHA was 26.5 μM TPF/g DM soil * 20 h.
